# Maternal stress and the MPOA: Activation of CRF receptor 1 impairs maternal behavior and triggers local oxytocin release in lactating rats

**DOI:** 10.1016/j.neuropharm.2018.02.019

**Published:** 2018-05-01

**Authors:** Stefanie M. Klampfl, Milena M. Schramm, Barbara M. Gaßner, Katharina Hübner, Audrey F. Seasholtz, Paula J. Brunton, Doris S. Bayerl, Oliver J. Bosch

**Affiliations:** a University of Regensburg, Regensburg, Germany; b University of British Columbia, Vancouver, BC, Canada; c Molecular and Behavioral Neuroscience Institute, University of Michigan, Ann Arbor, MI 48109-2200, USA; d Department of Biological Chemistry, University of Michigan, Ann Arbor, MI 48109-2200, USA; e Centre for Discovery Brain Sciences, University of Edinburgh, Edinburgh, UK

**Keywords:** Anxiety, Corticotropin-releasing factor, Corticotropin-releasing factor-binding protein, Maternal behavior, Medial preoptic area, Oxytocin

## Abstract

Maternal behavior and anxiety are potently modulated by the brain corticotropin-releasing factor (CRF) system postpartum. Downregulation of CRF in limbic brain regions is essential for appropriate maternal behavior and an adaptive anxiety response. Here, we focus our attention on arguably the most important brain region for maternal behavior, the hypothalamic medial preoptic area (MPOA).

Within the MPOA, mRNA for CRF receptor subtype 1 (protein: CRFR1, gene: *Crhr1*) was more abundantly expressed than for subtype 2 (protein: CRFR2, gene: *Crhr2*), however expression of *Crhr1*, *Crhr2* and CRF-binding protein (protein: CRFBP, gene: *Crhbp*) mRNA was similar between virgin and lactating rats. Subtype-specific activation of CRFR, predominantly CRFR1, in the MPOA decreased arched back nursing and total nursing under non-stress conditions. Following acute stressor exposure, only CRFR1 inhibition rescued the stress-induced reduction in arched back nursing while CRFR1 activation prolonged the decline in nursing. Furthermore, inhibition of CRFR1 strongly increased maternal aggression in the maternal defense test. CRFR1 activation had anxiogenic actions and reduced locomotion on the elevated plus-maze, however neither CRFR1 nor R2 manipulation affected maternal motivation. In addition, activation of CRFR1, either centrally or locally in the MPOA, increased local oxytocin release. Finally, inhibition of CRFBP (a potent regulator of CRFR activity) in the MPOA did not affect any of the maternal parameters investigated.

In conclusion, activity of CRFR in the MPOA, particularly of subtype 1, needs to be dampened during lactation to ensure appropriate maternal behavior. Furthermore, oxytocin release in the MPOA may provide a regulatory mechanism to counteract the negative impact of CRFR activation on maternal behavior.

## Highlights

• Activation of MPOA CRFR, predominantly CRFR1, impaired maternal care.
• Inhibition of MPOA CRFR1 strongly increased maternal aggression.
• Activation of MPOA CRFR1 triggered local oxytocin release.
• CRF-binding protein in the MPOA did not regulate maternal behavior.
• Dampened CRFR1 activity in MPOA is vital to ensure appropriate maternal behavior.

## 1 Introduction

The display of appropriate maternal behavior is the result of a variety of peripartum adaptations, including activation and inhibition of specific neurotransmitter systems in the brain. While oxytocin and vasopressin typically promote maternal behavior, and are hence up-regulated postpartum (e.g. Bosch and Neumann, 2008, 2012; Pedersen et al., 1994; van Leengoed et al., 1987), the corticotropin-releasing factor (CRF) system impedes maternal behavior and thus needs to be down-regulated (Gammie et al., 2004; Klampfl et al., 2013, Klampfl et al., 2014, Klampfl et al., 2016a, Klampfl et al., 2016b).

The CRF system consists of four ligands, CRF (protein: CRF, gene: *Crh*) and the urocortins 1–3, which bind with different affinities to the two CRF receptors (protein: CRFR, gene: *Crhr*): CRFR1 and CRFR2 (Reul and Holsboer, 2002a). In addition, activation of the CRFR is regulated and, for the most part attenuated, by the secretory glycoprotein CRF-binding protein (protein: CRFBP, gene: *Crhbp*) (Seasholtz et al., 2002; Sutton et al., 1995). CRF is the major stress neuropeptide involved in cellular, neuroendocrine, and behavioral responses to stress (Bale and Vale, 2004; Vale et al., 1981). It is released upon stressor exposure and triggers the central and peripheral stress response. Furthermore, CRF has anxiogenic and pro-depressive actions, amongst others (Reul and Holsboer, 2002a, b), which makes the CRF system one of the most promising candidate systems for treating mood disorders such as anxiety and depression.

During the postpartum period, activation of central CRFR strongly impairs maternal care and maternal aggression thereby inducing maternal neglect in lactating rats (Klampfl et al., 2013, 2014, 2016a), mice (D'Anna and Gammie, 2009; D'Anna et al., 2005; Gammie et al., 2004), and marmoset monkeys (Saltzman et al., 2011). These effects are observed not only by central activation of CRFR via intracerebroventricular (ICV) administration of CRFR agonists, but also by local stimulation of the CRFR1 and CRFR2 subtypes in the lateral septum (D'Anna and Gammie, 2009; D'Anna et al., 2005) and bed nucleus of the stria terminalis (BNST) (Klampfl et al., 2014, Klampfl et al., 2016a).

The BNST and the adjacent medial preoptic area (MPOA), together form the maternal ‘super-region’ for regulating maternal behavior (Numan and Insel, 2003; Numan and Stolzenberg, 2009). Given that the CRF family of neuropeptides act in both the anterior and posterior BNST to influence maternal behavior (Klampfl et al., 2014, 2016a), it seems intuitive that the CRF system may also play a role in the counterpart of the maternal super-region, the MPOA. Indeed, it is well established that the MPOA is crucial for the onset and maintenance of maternal behavior, especially maternal care and maternal motivation (Numan and Stolzenberg, 2009). Here, the nonapeptide oxytocin has been shown to promote maternal behavior in several different species (Numan and Insel, 2003). Oxytocinergic projections from the paraventricular nucleus are thought to supply the MPOA with constantly high concentrations of oxytocin during lactation (Bosch and Neumann, 2012). In addition, oxytocin receptors are substantially up-regulated in the MPOA peripartum (Meddle et al., 2007), which facilitates finely-tuned regulation of maternal care even under constant levels of intracerebral oxytocin throughout mother-pup interactions (Bosch and Neumann, 2012). Importantly, oxytocin directly interacts with the CRF system, as oxytocin receptors are expressed on CRF neurons in the BNST and CRFR2 are expressed on oxytocin neurons in the paraventricular nucleus (Dabrowska et al., 2013), indicating a reciprocal neuromodulatory role for both peptides.

In the present study, we hypothesized that like the BNST, the MPOA CRF system is also involved in the regulation of maternal behavior. Hence, we assessed the expression profiles of both CRFR and CRFBP in the MPOA and examined the effects of modulating CRFR or CRFBP in the MPOA under stress and non-stress conditions. Furthermore, as one of the main mediators of maternal behavior in the MPOA is the oxytocin system (Bosch and Neumann, 2012), we hypothesized that local oxytocin release is affected by changes in CRFR signaling.

## 2 Materials & methods

### 2.1 Animals and housing

Virgin female Sprague-Dawley rats (220–250 g; Charles River Laboratories, Sulzfeld, Germany) were housed under standard laboratory conditions (change of bedding once per week, RT 22 ± 2 °C, 55% relative humidity, 12:12 h light/dark cycle, lights on at 6 a.m.) with access to water and standard rat chow *ad libitum*. All rats were initially housed in groups of 3–4 (for further details see below). Female rats were mated with sexually experienced male rats and pregnancy was confirmed by the presence of sperm in vaginal smears (pregnancy day (PD) 1). A separate, naïve cohort of rats was used for each experiment, and in each case rats were randomly assigned to the different treatment groups.

For experiment 1, virgin and lactating females were treated similarly; virgins were single-housed 7 days prior to brain collection, consistent with the single-housing period of the lactating rats, which were single-housed on PD 18 and killed 7 days later (lactation day (LD) 4). In experiments 2 and 5, females underwent surgery on PD 18 and were subsequently single-housed to guarantee recovery and undisturbed parturition (Klampfl et al., 2013). In experiments 3 and 4, rats were single-housed on PD 18 and underwent surgery on LD 1. On the day of birth, litters of all dams were culled to eight pups of mixed sexes. All rats were handled twice daily on PD 16–17 and during the single-housing period (except on the day of surgery and birth) to reduce non-specific stress responses during the experiments (Neumann et al., 1998).

For the maternal defense test, naïve virgin female Wistar rats (200–220 g; Charles River Laboratories) were used as intruders at random stages of their estrous cycle. Intruder rats were kept group-housed in a separate room to avoid olfactory recognition (Bosch, 2013).

The experiments were approved by the Committee on Animal Health and Care of the local government and conformed with the European Directive (2010/63/EU) on the ethical use of animals. All reasonable efforts were made to minimize the number of rats used and their suffering.

### 2.2 Behavioral tests

All tests were performed between 8 a.m. and 3 p.m. during the light phase of the cycle. In experiments with repeated drug infusion (experiments 2 and 5), rats received the same drug throughout the experiment. After infusion, dams were immediately returned to their home cage.

#### 2.2.1 Maternal care

Maternal care was monitored before and after drug infusion under non-stress and stress conditions (maternal defense test) (Klampfl et al., 2013, 2014, 2016a, b). Observations were made for 10 s every 2nd min in 30 min blocks according to an established protocol (Bosch and Neumann, 2008). The main parameter for the quality of maternal care was the occurrence of arched back nursing (ABN) (Bosch, 2011), an active nursing posture where the dam is engaged in a quiescent kyphosis (Stern and Johnson, 1990). Other behavioral parameters scored were hovering over the pups and blanket nursing posture, which together with ABN were counted as total nursing, thereby indicating the quantity of maternal care (Klampfl et al., 2014). Pup retrieval/mouthing and licking/grooming (LG) were also scored. Additionally, non-maternal behaviors were quantified, i.e. locomotion (including digging/burrowing and cage exploration), self-grooming, and sleeping/resting, which were summed up and are presented as ‘off-nest behavior’.

#### 2.2.2 Maternal motivation

The dams' maternal motivation was tested in the modified pup retrieval test (PRT) as described previously (Bayerl et al., 2016). Briefly, dams were habituated to a red Perspex house for 150 min the day prior to the PRT. On the day of testing, the pups were separated from the dam for 60 min, during which the red house was re-introduced to the dam's cage. Afterwards, the house and the dam were transferred to a plastic testing box (54 cm × 34 cm × 31 cm), which contained some bedding and the pups. Here, the number of pups retrieved into the house within the 15-min testing period was counted as well as the latencies to retrieve the first and the last pup.

#### 2.2.3 Maternal aggression

To assess maternal aggression, the maternal defense test was performed in a separate room, to which the dams were transported 60 min prior to the test. After drug infusion, the lactating resident (in the presence of the litter) was confronted with an unknown virgin female intruder in her home cage for 10 min as described previously (Bosch et al., 2005; Neumann et al., 2001). The dam's behavior was videotaped for subsequent analysis by an experienced observer blind to the treatment using JWatcher (http://www.jwatcher.ucla.edu/). The following behavioral parameters were scored: total number of attacks, latency to first attack, lateral threat, keep down, and offensive upright as well as non-aggressive behaviors (for detailed description see (Bosch, 2013)).

#### 2.2.4 Anxiety-related behavior

Anxiety-related behavior was tested on the elevated plus-maze (EPM) as previously described (Neumann et al., 2000; Pellow et al., 1985). Briefly, the plus-shaped maze consists of two open arms (50 cm × 10 cm, 80 lux) and two closed arms (50 cm × 10 cm x 30 cm, 10 lux) surrounding a neutral square-shaped central zone (10 cm × 10 cm, 65 lux) and is elevated 82 cm above the floor. After drug infusion, the rats were placed in the neutral zone to freely explore the maze for 5 min. Both fore-paws needed to be on an open arm to be counted as open arm time. All four paws needed to be in an open arm to be counted as full open arm entry. The percentage of time spent on the open arm versus all areas (open arm, closed arm, and neutral zone) and the number of full open arm entries were indicators for anxiety-related behavior. The number of closed arm entries was used to measure locomotion (Neumann et al., 2000).

### 2.3 Experimental design

#### 2.3.1 Experiment 1: Expression of *Crhr* and *Crhbp* mRNA in the MPOA of virgin and lactating rats

To compare the effect of reproductive status on *Crhr1, Crhr2,* and *Crhbp* mRNA expression in the MPOA, 29 virgin and lactating rats were killed in the morning of LD 4 (*Crhr* mRNA), LD 7 (*Crhbp* mRNA) or equivalent in virgin rats, i.e. after 7 days of single-housing under basal conditions by conscious decapitation as anesthesia can affect central peptide content as well as activate the HPA axis (Bekhbat et al., 2016; Smagin and Goeders, 2004; van Duinen et al., 2005). The brains were rapidly removed, flash frozen in *n*-methylbutane on dry ice, and stored at −20 °C until further processing. Later, frozen brains were sectioned at 16 μm using a cryostat (Model CM3050S Leica Microsystems GmbH, Nussloch, Germany), mounted on polysine slides, and stored at −20 °C until further processing.

*Crhr1*, *Crhr2,* and *Crhbp* mRNA *in situ* hybridization was performed using an established protocol with previously described cRNA probes for *Crhr1*, *Crhr2* (Brunton et al., 2009, 2011), and *Crhbp* (Speert et al., 2002; Stinnett et al., 2015). Autoradiograms of the MPOA (Bregma −0.24 mm to −0.6 mm (Paxinos and Watson, 1998)) were analyzed with ImageJ (V1.46, NIH image software) as previously described (Brunton et al., 2011). Measurements were made bilaterally over six sections per rat. Brain sections hybridized with ^35^S-UTP-labeled cRNA sense probes (negative controls) showed no signal above background.

#### 2.3.2 Experiments 2 and 5: Pharmacological manipulation of CRFR and CRFBP in the MPOA of lactating rats

On PD 18, 54 females (experiment 2: n = 34; experiment 5: n = 20) were implanted bilaterally with 23 G guide cannula targeting the MPOA (0.4 mm caudal, 0.8 mm lateral, 6.8 mm ventral to bregma (Paxinos and Watson, 1998)) under inhalation anesthesia (Isoflurane; Baxter Germany GmbH, Unterschleiβheim, Germany) and sterile conditions as described earlier (Bosch et al., 2010). Substances were infused using a 27 G infusion cannula. In experiment 2, lactating rats received one of the following treatments 10 min before testing: (i) VEH (0.5 μl of sterile Ringer's solution + 4% DMSO; pH 7.4; B. Braun Melsungen, Melsungen, Germany), (ii) CRFR1 agonist, human/rat CRF (h/rCRF; 1 μg/0.5 μl; Tocris Bioscience, Ellisville, Missouri, USA), (iii) CRFR1 specific antagonist, CP-154,526 (0.4 μg/0.5 μl; Tocris Bioscience), (iv) CRFR2 specific agonist, human Ucn 3 (hUcn3; also known as stresscopin; 3 μg/0.5 μl; Phoenix Pharmaceuticals, Karlsruhe, Germany), or (v) CRFR2 specific antagonist (astressin-2B; 4 μg/0.5 μl; Sigma-Aldrich, Steinheim, Germany). In experiment 5, lactating rats received either (i) VEH (0.5 μl of sterile Ringer's solution; pH 7.4; B. Braun Melsungen) or (ii) the CRFBP inhibitor CRF_(6-33)_ (5 μg/0.5 μl; Bachem, Bubendorf, Switzerland) 20 min before testing. Doses and the lag time between the infusion and behavioral testing were chosen based on previous studies (Klampfl et al., 2014, Klampfl et al., 2016a, Klampfl et al., 2016b; Zorrilla et al., 2001).

Maternal care was observed in the same rats under non-stress conditions (LD 1) and stress conditions (LD 7) in their home cage in the colony room. Under non-stress conditions, dams were observed from 8 a.m.–9 a.m., followed by treatment infusion and subsequent observation for a further 120 min. In addition, 5 h after the infusion (2 p.m.–3 p.m.), maternal care was monitored again to assess any potential long-lasting effects of the drug treatment. On LD 7, dams were observed from 8 a.m.–9 a.m. in their colony room before being moved to the test room. At 10 a.m., dams were VEH/drug-infused, exposed to stress using the maternal defense test and immediately afterwards returned to the colony room, where maternal care was observed for a further 60 min to assess the effects of the preceding stressor on maternal care. Additionally, maternal motivation (LD 3), anxiety-related behavior (LD 5), and maternal aggression (LD 7) were assessed as described in 2.2.

#### 2.3.3 Experiment 3: Pharmacological ICV CRFR activation and simultaneous microdialysis in the MPOA of lactating rats

On LD 1, 22 females were implanted with a 21 G guide cannula targeting the left lateral ventricle (1.0 mm caudal, 1.6 mm lateral, 2.1 mm ventral to bregma (Paxinos and Watson, 1998)) and a microdialysis probe (molecular cut-off: 18 kDa; Hemophan, Gambro Dialysatoren, Hechingen, Germany) targeting the right MPOA (0.4 mm caudal, 0.9 mm lateral, 8.8 mm ventral to bregma (Paxinos and Watson, 1998)) under inhalation anesthesia (Isoflurane; Baxter Germany GmbH) and sterile conditions as described earlier (Bosch et al., 2010).

On LD 3, the inflow adapter of the microdialysis probe was connected via PE-20 tubing to a syringe mounted onto a microinfusion pump. The outflow adapter was attached to a 1.5 ml collection tube containing 10 μl 0.1 N HCl. The probe was flushed at a rate of 3.3 μl/min with sterile Ringer's solution (pH 7.4) for 90 min before 30 min sample collections commenced. Starting at 10 a.m., samples 1 and 2 were collected under basal conditions. Afterwards, dams were infused ICV with either (i) VEH (5 μl sterile Ringer's solution + 4% DMSO, pH 7.4), (ii) CRFR1 agonist h/rCRF (1 μg/5 μl), or (iii) CRFR2 specific agonist hUcn3 (3 μg/5 μl). Samples 3–5 were collected during the 90-min period following drug injection. Additionally, maternal care was observed throughout the entire sampling period. All microdialysates were immediately frozen on dry ice and stored at −80 °C until quantification of oxytocin (see 2.4).

#### 2.3.4 Experiment 4: Intra-MPOA CRFR1 agonist retrodialysis in lactating rats

Separate groups of lactating rats (n = 14) were implanted unilaterally with a microdialysis probe targeting the right MPOA on LD 1. Microdialysis was performed on LD 3 as described in 2.3.3 for experiment 3, except that the perfusion rate was 1 μl/min (Bosch et al., 2005).

Starting at 10 a.m., samples 1 and 2 were collected under basal conditions 30 min apart. Next, the syringes containing Ringer's solution were swapped for those filled with either (i) VEH (sterile Ringer's solution + 4% DMSO, pH 7.4) or (ii) CRFR1 agonist h/rCRF (0.05 μg/μl/min) for 30 min (sample 3). Syringes were then switched back to those containing sterile Ringer's solution and samples 4 and 5 were collected 30 min apart. Microdialysate samples were immediately frozen on dry ice and stored at −80 °C until quantification of oxytocin (see 2.4).

### 2.4 Radioimmunoassay for oxytocin in microdialysates

Oxytocin peptide content was measured in lyophilized dialysates by a highly sensitive and selective radioimmunoassay (detection limit: 0.1 pg/sample; cross reactivity of the antisera with other related peptides < 7%; RIAgnosis, Munich, Germany; for details see (Landgraf et al., 1995)).

### 2.5 Histology

All rats were killed at the end of the experiments (experiment 1: decapitation without anesthesia; experiments 2 and 5: LD 7, CO_2_ overdose; experiment 3: LD 4, CO_2_ overdose; experiment 4: LD 3, CO_2_ overdose), and brains were collected and flash frozen in *n*-methylbutane for histological verification of correct cannula placement. To identify implantation sites, 0.5 μl ink was infused post-mortem into the brain via the cannula (experiments 2 and 3; Pelikan Ink 4001, Hanover, Germany; diluted 1:20 in Ringer's solution). Brains were then removed, flash frozen, cut in 40 μm coronal sections, and mounted on slides. Brains were checked for potential diffusion outside the MPOA according to an established protocol (Klampfl et al., 2014, 2016a, b) and were excluded from analysis if any staining was detected beyond the MPOA. Brains from experiments 3 and 4 were removed, flash frozen, sectioned, and mounted on slides. Brain sections from all experiments were Nissl stained to locate the tip of the infusion cannula/microdialysis probe. Only rats identified as having correctly positioned implants were included in the statistical analysis.

### 2.6 Statistical analysis

Data were analyzed using either an independent *t*-test, paired *t*-test, two-way ANOVA (factors: brain site, reproductive status), or two-way repeated measures (RM) ANOVA (factors: time x treatment) followed by Fisher's LSD *post hoc* test. For all statistical analyses, the software package SPSS 24.0 (IBM, Armonk, NY, USA) was used. Data are presented as group means ± SEM, and p ≤ 0.05 was considered statistically significant.

## 3 Results

### 3.1 Experiment 1: Expression of *Crhr1, Crhr2,* and *Crhbp* mRNA in the MPOA of virgin and lactating rats

**Fig. 1:**
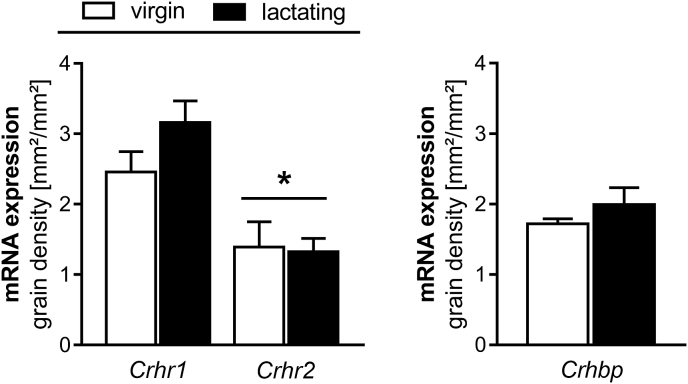
***Crhr1, Crhr2* (left) and *Crhbp* (right) mRNA expression in the MPOA in virgin and lactating rats.** Data are presented as mean grain area + SEM. n = 5–10 per group. *p < 0.05 versus *Crhr1* (two-way ANOVA; factors: reproductive status, brain site).

*Crhr1* and *Crhr2* mRNA expression in the MPOA did not differ between virgin and lactating rats (Fig. 1 left); however, levels of *Crhr1* mRNA expression were significantly greater than *Crhr2* mRNA, independent of reproductive status (two-way ANOVA; factor: receptor subtype; F_1,20_ = 24.66, p < 0.01; Fig. 1 left). There were no differences in *Crhbp* mRNA expression in the MPOA between virgin and lactating rats (Fig. 1 right).

### 3.2 Experiment 2: Pharmacological manipulation of CRFR in the MPOA of lactating rats

#### 3.2.1 Maternal care under non-stress conditions

**Fig. 2:**
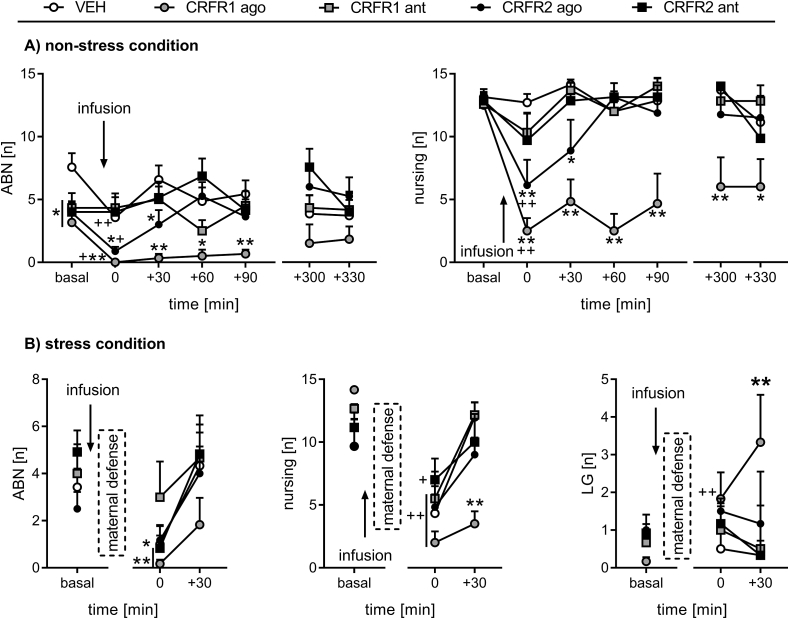
**Effect of pharmacological manipulation of MPOA CRFR1 or CRFR2 on maternal care under (A) non-stress conditions on LD 1 or (B) stress conditions on LD 7.** Arched back nursing (ABN), total nursing, and licking/grooming (LG) were scored for 60 min before (basal) and (A) for 90 min after infusion (t 0 to t +90) as well as for 60 min in the afternoon (t +300 to t +330) or (B) for 60 min after infusion combined with the maternal defense test (t 0 to t +30). Dams received an acute bilateral infusion of either (i) vehicle (VEH), (ii) CRFR1 agonist (ago; h/rCRF), (iii) CRFR1 antagonist (ant; CP-154,526), (iv) CRFR2 ago (hUcn3/stresscopin), or (v) CRFR2 ant (astressin-2B) into the MPOA. Data are presented as group means + SEM. n = 6–8 rats per group. **p ≤ 0.01, *p ≤ 0.05 versus VEH-treated group; ++ p ≤ 0.01, + p ≤ 0.05 versus basal levels in the same group (two-way RM ANOVA).

*ABN*: ABN significantly differed over time between the groups (two-way RM ANOVA; F_24,174_ = 1.81, p = 0.01; Fig. 2A left). Compared to basal levels, ABN was decreased at t 0 min in the VEH (p < 0.01) and both agonist groups (CRFR1 agonist: p = 0.03; CRFR2 agonist: p = 0.01), but not the antagonist-treated groups. ABN was significantly lower in the CRFR1 agonist-treated dams after infusion, i.e. at t 0 min, t +30 min, t +60 min, and t +90 min, compared with the VEH-treated group (p < 0.01, in each case). Furthermore, ABN was lower in the CRFR2 agonist-treated dams at t 0 min and t +30 min (p = 0.02, in each case) compared to the VEH group. No differences were found between the antagonist-treated groups and the VEH group at any time points following the drug infusion.

*Nursing*: Nursing differed over time between the groups (two-way RM ANOVA; F_24, 174_ = 1.89, p = 0.01; Fig. 2A right). Compared to basal levels, nursing was decreased at t 0 min in both agonist-treated groups (p < 0.01, in each case). Following the infusion, nursing was persistently lower in the CRFR1 agonist-treated dams (t 0 min – t +300 min: p < 0.01, t +330 min: p = 0.05), compared with the VEH group. Nursing was also lower in the CRFR2 agonist group, compared with the VEH group, however the effect was more transient (t 0 min, p < 0.01; t +30 min, p = 0.02). The amount of nursing in the antagonist-treated dams did not differ from the control rats.

*Other maternal behavior*: No significant differences were observed in other maternal behaviors, i.e. pup retrieval/mouthing and LG (data not shown).

*Non-maternal behavior*: Significant interactions were found for overall off-nest behaviors (two-way RM ANOVA; F_24,174_ = 1.78, p = 0.02), locomotion (F_24,174_ = 1.79, p = 0.01), and self-grooming (F_24,174_ = 4.01, p < 0.01; Table 1). Both the CRFR1 and CRFR2 agonist-treated dams showed more off-nest behavior (p < 0.01, in each case), locomotion (CRFR1 agonist: p < 0.01; CRFR2 agonist: p = 0.01), and self-grooming (p < 0.01, in each case) at t 0 min compared to basal. Also, the CRFR2 antagonist-treated rats exhibited more off-nest behavior at t 0 min compared to basal (p = 0.04). Compared to the VEH-treated dams, the CRFR1 agonist-treated dams displayed more off-nest behavior from t 0 min to t +300 min, locomotion from t 0 min to t +330 min, and self-grooming from t 0 min to t +90 min (p < 0.01, in each case). The CRFR2 agonist-treated dams exhibited more off-nest behavior at t 0 min (p < 0.01) and t +30 min (p = 0.01), compared with the VEH group. There was no significant effect of the CRFR1 antagonist on any of the non-maternal behaviors analyzed under non-stress conditions and no significant differences were detected for sleeping/resting between any of the treatment groups (Table 1).

**Table 1:** Effect of pharmacological manipulation of MPOA CRFR1 or CRFR2 on non-maternal behaviors under non-stress conditions on LD 1.

Behavior	Group	Occurrence [n]						
		Basal	0 min	+30 min	+60 min	+90 min	+300 min	+330 min
Off-nest	VEH	1.1 ± 0.5	1.6 ± 0.8	0.1 ± 0.1	2.1 ± 1.2	1.7 ± 1.5	0.9 ± 0.5	2.7 ± 2.0
	CRFR1 ago	1.5 ± 0.7	11.8 ± 1.0**^++^	7.8 ± 2.3**	9.7 ± 2.6**	8.8 ± 2.8**	7.8 ± 2.7**	7.8 ± 2.2
	CRFR1 ant	1.3 ± 0.4	3.3 ± 1.5	0.8 ± 0.6	1.8 ± 0.8	0.5 ± 0.3	1.2 ± 0.6	1.3 ± 0.8
	CRFR2 ago	0.7 ± 0.2	8.9 ± 2.0**^++^	6.0 ± 2.5*	1.4 ± 0.6	0.9 ± 0.3	2.9 ± 2.0	2.6 ± 1.8
	CRFR2 ant	1.0 ± 0.5	4.7 ± 2.2^+^	1.4 ± 0.6	1.1 ± 1.0	0.1 ± 0.1	0.1 ± 0.1	3.6 ± 1.8
Locomotion	VEH	0.6 ± 0.4	1.0 ± 0.6	0.0 ± 0.0	0.9 ± 0.4	1.0 ± 0.8	0.4 ± 0.3	0.3 ± 0.2
	CRFR1 ago	0.2 ± 0.1	7.3 ± 1.1**^++^	4.5 ± 1.6**	6.5 ± 1.6**	5.8 ± 2.1**	5.8 ± 2.5**	6.5 ± 2.0**
	CRFR1 ant	0.7 ± 0.3	2.5 ± 1.3	0.2 ± 0.2	1.2 ± 0.4	0.2 ± 0.2	0.2 ± 0.2	0.8 ± 0.3
	CRFR2 ago	0.4 ± 0.1	2.8 ± 0.7^+^	1.4 ± 0.7	0.5 ± 0.3	0.9 ± 0.3	0.0 ± 0.0	0.1 ± 0.1
	CRFR2 ant	0.6 ± 0.3	1.4 ± 0.7	0.4 ± 0.3	0.9 ± 0.7	0.1 ± 0.1	0.1 ± 0.1	1.0 ± 0.4
Self-grooming	VEH	0.3 ± 0.1	0.4 ± 0.3	0.1 ± 0.1	0.4 ± 0.3	0.0 ± 0.0	0.0 ± 0.0	0.3 ± 0.2
	CRFR1 ago	0.2 ± 0.1	3.5 ± 0.8**^++^	2.7 ± 0.5**	1.7 ± 0.6**	1.1 ± 0.5**	0.5 ± 0.2	0.5 ± 0.5
	CRFR1 ant	0.6 ± 0.2	0.6 ± 0.3	0.5 ± 0.3	0.3 ± 0.2	0.3 ± 0.3	0.3 ± 0.3	0.0 ± 0.0
	CRFR2 ago	0.2 ± 0.1	1.4 ± 0.4^++^	0.3 ± 0.2	0.0 ± 0.0	0.0 ± 0.0	0.3 ± 0.2	0.5 ± 0.3
	CRFR2 ant	0.1 ± 0.1	0.7 ± 0.4	0.3 ± 0.2	0.0 ± 0.0	0.0 ± 0.0	0.0 ± 0.0	1.0 ± 0.5
Sleeping/ resting	VEH	0.0 ± 0.0	0.0 ± 0.0	0.0 ± 0.0	0.4 ± 0.4	0.0 ± 0.0	0.4 ± 0.4	2.1 ± 2.1
	CRFR1 ago	0.0 ± 0.0	0.0 ± 0.0	0.0 ± 0.0	0.2 ± 0.2	0.8 ± 0.8	0.0 ± 0.0	0.0 ± 0.0
	CRFR1 ant	0.0 ± 0.0	0.0 ± 0.0	0.0 ± 0.0	0.0 ± 0.0	0.0 ± 0.0	0.7 ± 0.7	0.0 ± 0.0
	CRFR2 ago	0.0 ± 0.0	4.6 ± 2.0	4.4 ± 2.2	0.6 ± 0.6	0.0 ± 0.0	2.6 ± 1.8	1.9 ± 1.9
	CRFR2 ant	0.0 ± 0.0	2.3 ± 1.5	0.6 ± 0.6	0.0 ± 0.0	0.0 ± 0.0	0.0 ± 0.0	0.6 ± 0.6

The occurrence of all off-nest behaviors was scored for 60 min before (averaged as basal) and 90 min after drug infusion (immediately before t 0 min), as well as during an additional 60 min period in the afternoon. Off-nest behavior is further divided into locomotion (including digging/burrowing and any explorative behavior in the home cage), self-grooming, and sleeping/resting. For details on treatments, see the Fig. 2 legend. Data are presented as group means ± SEM. n = 6–8 rats per group. **p ≤ 0.01, *p ≤ 0.05 versus VEH; ++ p ≤ 0.01, + p ≤ 0.05 versus basal.

#### 3.2.2 Maternal care under stress conditions

*ABN*: There was a significant effect of time (two-way RM ANOVA; F_2,50_ = 9.72, p < 0.01; Fig. 2B left), but not of treatment. Separate statistics (paired *t*-test) showed that ABN decreased after the infusion/maternal defense test in all groups (VEH: p = 0.05; CRFR1 agonist, CRFR2 antagonist: p < 0.01, in each case), except the CRFR1 antagonist- (p = 0.7) and CRFR2 agonist-treated dams (p = 0.1) at t 0 min versus basal.

*Nursing*: Nursing significantly changed over time between the groups (two-way RM ANOVA; F_8,50_ = 4.48, p < 0.01; Fig. 2B middle). Stress exposure (maternal defense test) triggered a reduction in nursing in all groups at t 0 min compared to basal levels (VEH, CRFR1 agonist, CRFR1 antagonist: p < 0.01, in each case; CRFR2 agonist: p = 0.01; CRFR2 antagonist: p = 0.02), which had recovered by t +30 min in all groups except for the CRFR1 agonist-treated dams (p < 0.01 vs. VEH).

*Other maternal behavior*: LG differed over time between the groups (two-way RM ANOVA; F_8,50_ = 2.58, p = 0.01; Fig. 2B right). The CRFR1 agonist-treated rats showed increased LG at t 0 min (p < 0.01) compared to basal levels and at t +30 min (p < 0.01) compared to the VEH group. No effect was found for pup retrieval/mouthing (data not shown).

*Non-maternal behavior*: Significant interactions were found for overall off-nest behavior (two-way RM ANOVA; F_8,50_ = 2.05, p = 0.05) and self-grooming (F_8,50_ = 2.29, p = 0.03; Table 2). All groups except the CRFR2 antagonist-treated dams showed more off-nest behavior at t 0 min compared to basal levels (VEH, CRFR1 agonist, CRFR1 antagonist: p < 0.01, in each case; CRFR2 agonist: p = 0.03). In addition, the CRFR1 agonist- and CRFR1 antagonist-treated rats exhibited significantly more self-grooming at t 0 min compared to basal conditions (CRFR1 agonist: p < 0.01; CRFR1 antagonist: p = 0.01). The CRFR1 agonist-treated dams showed more off-nest behavior at t +30 min (p = 0.02) and self-grooming at t 0 min and t +30 min (p < 0.01, in each case). No other changes were detected in any of the treatment groups.

**Table 2:** Effect of pharmacological manipulation of MPOA CRFR1 or CRFR2 on non-maternal behaviors under stress conditions on LD 7.

Behavior	Group	Occurrence [n]		
		Basal	0 min	+30 min
Off-nest	VEH	4.7 ± 2.2	10.2 ± 1.8^++^	2.7 ± 1.2
	CRFR1 ago	0.5 ± 0.2	11.0 ± 1.0^++^	8.0 ± 1.4*
	CRFR1 ant	1.3 ± 0.2	8.7 ± 2.3^++^	2.0 ± 1.1
	CRFR2 ago	4.5 ± 2.4	8.8 ± 2.0^+^	4.7 ± 1.7
	CRFR2 ant	2.9 ± 1.2	6.5 ± 2.2	4.7 ± 2.3
Locomotion	VEH	1.3 ± 0.7	3.5 ± 1.8	0.7 ± 0.5
	CRFR1 ago	0.1 ± 0.1	4.3 ± 0.7	3.0 ± 0.7
	CRFR1 ant	0.2 ± 0.1	3.2 ± 0.7	0.0 ± 0.0
	CRFR2 ago	0.4 ± 0.3	4.0 ± 1.6	1.7 ± 0.6
	CRFR2 ant	0.6 ± 0.2	2.8 ± 0.9	0.8 ± 0.3
Self-grooming	VEH	0.7 ± 0.2	1.5 ± 0.6	0.5 ± 0.3
	CRFR1 ago	0.3 ± 0.1	5.0 ± 0.8**^++^	2.5 ± 1.3**
	CRFR1 ant	0.2 ± 0.1	2.0 ± 0.9^+^	0.0 ± 0.0
	CRFR2 ago	0.8 ± 0.4	2.2 ± 0.8	0.7 ± 0.3
	CRFR2 ant	0.5 ± 0.2	1.5 ± 0.4	1.0 ± 0.6
Sleeping/ resting	VEH	1.0 ± 1.0	3.5 ± 2.2	1.3 ± 1.3
	CRFR1 ago	0.0 ± 0.0	0.5 ± 0.5	0.5 ± 0.5
	CRFR1 ant	0.3 ± 0.3	1.8 ± 1.3	1.7 ± 1.0
	CRFR2 ago	1.9 ± 1.3	0.0 ± 0.0	1.5 ± 1.5
	CRFR2 ant	0.4 ± 0.3	1.7 ± 1.7	2.5 ± 2.5

The occurrence of all off-nest behaviors was scored for 60 min before (averaged as basal) and 60 min after the infusion combined with the maternal defense test (immediately before t 0 min). Off-nest behavior is further divided into locomotion (including digging/burrowing and any explorative behavior in the home cage), self-grooming, and sleeping/resting. For details on treatments, see the Fig. 2 legend. Data are presented as group means ± SEM. n = 6 rats per group. **p ≤ 0.01, *p ≤ 0.05 versus VEH; ++ p ≤ 0.01, + p ≤ 0.05 versus basal.

#### 3.2.3 Maternal motivation

None of the CRFR manipulations affected maternal motivation in the PRT (data not shown).

#### 3.2.4 Maternal aggression

**Fig. 3:**
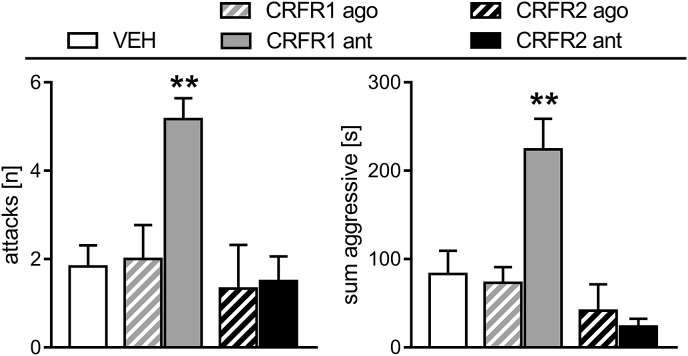
**Effect of pharmacological manipulation of MPOA CRFR1 or CRFR2 on maternal aggression in lactating rats.** Maternal aggression against a virgin female intruder was scored during a 10-min maternal defense test. The number of attacks (left) and sum of aggressive behaviors (right) exhibited by the resident are shown. For details on treatments, see the Fig. 2 legend. Data are presented as mean + SEM. n = 6 per group. **p < 0.01 versus VEH (one-way ANOVA; factor: treatment).

The number of attacks (one-way ANOVA; F_4,29_ = 5.36, p < 0.01; Fig. 3 left) and the sum of aggressive behaviors (F_4,29_ = 9.89, p < 0.01; Fig. 3 right) differed between the groups. Both measures for maternal aggressive behavior were significantly greater in the CRFR1 antagonist-treated dams (p < 0.01, in each case), compared with all other groups.

#### 3.2.5 Anxiety-related behavior

**Fig. 4:**
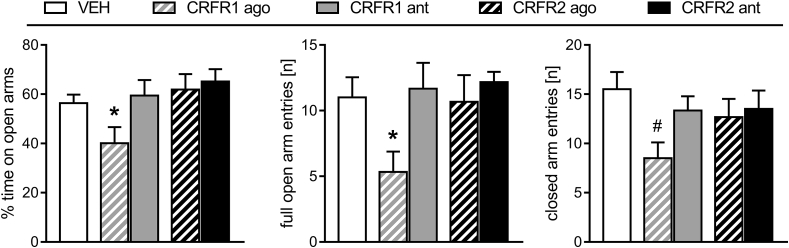
**Effect of pharmacological manipulation of MPOA CRFR1 or CRFR2 on anxiety-related behavior in lactating rats.** The percent time spent on the open arms, the number of full open arm entries and closed arm entries during the 5-min test are shown. For details on treatments, see the Fig. 2 legend. Data are presented as group means + SEM. n = 6 per group. *p < 0.05 versus VEH (one-way ANOVA; factor: treatment); #p < 0.05 versus VEH (independent *t*-test).

On the EPM, the percentage of time spent on the open arms (one-way ANOVA; F_4,29_ = 2.99, p = 0.04; Fig. 4 left) and the number of full open arm entries (F_4,29_ = 2.83, p = 0.05; Fig. 4 middle) significantly differed between the groups. The CRFR1 agonist-treated dams spent significantly less time on the open arms (p = 0.05) and made fewer full open arm entries (p = 0.02) compared with the VEH-treated rats. Entries onto the closed arms (indicating locomotor activity) did not differ between groups when data was analyzed using a one-way ANOVA; however separate statistical analysis revealed that the CRFR1 agonist-treated dams made fewer closed arm entries indicating decreased locomotor activity compared to the VEH-treated dams (independent *t*-test; t_10_ = 2.93, p = 0.02; Fig. 4 right).

### 3.3 Experiment 3: Effects of ICV CRFR activation on maternal care and intra-MPOA oxytocin release in lactating rats

#### 3.3.1 Maternal care

**Fig. 5:**
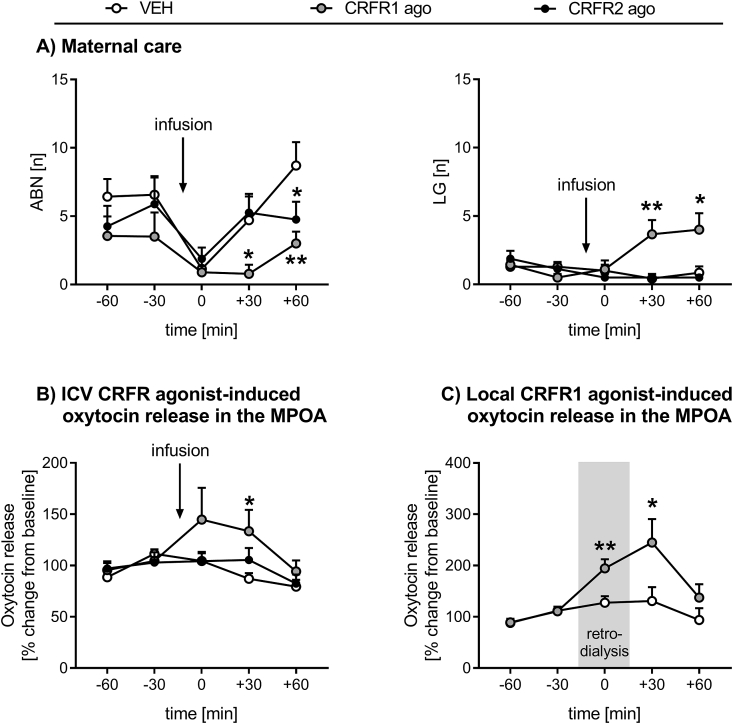
**Effect of ICV or intra-MPOA CRFR activation on maternal care and oxytocin release in the MPOA of lactating rats.** Arched back nursing (ABN) and licking/grooming (LG) (A) as well as oxytocin release in the MPOA (B) were assessed for 60 min before (t −60 to t −30) and 90 min after ICV drug infusion (t 0 to t +60) under non-stress conditions. (C) Oxytocin release in the MPOA was measured for 60 min before (t −60 to t −30), 30 min during (t 0), and 60 min after (t +30 to t +60) retrodialysis. For (A) and (B), dams received an acute ICV infusion of either (i) vehicle (VEH), (ii) CRFR1 agonist (ago; h/rCRF), or (iii) CRFR2 ago (hUcn3/stresscopin). For (C), dams received chronic CRFR1 agonist retrodialysis into the MPOA. Data are presented as group means + SEM. n = 6–8 rats per group. **p ≤ 0.01, *p ≤ 0.05 versus VEH-treated group (two-way RM ANOVA).

*ABN*: There was a significant effect of time (two-way RM ANOVA; F_4,80_ = 5.60, p < 0.01) and treatment (F_2,20_ = 4.27, p = 0.02; Fig. 5A left) on ABN. The CRFR1 agonist-treated dams performed significantly less ABN after ICV drug infusion at t +30 min (p = 0.05) and t +60 min compared to the VEH-treated dams (p = 0.01). Additionally, the CRFR2 agonist-treated dams displayed less ABN at t +60 min compared to the VEH group (p = 0.04).

*Nursing*: Nursing differed depending on time (two-way RM ANOVA; F_4,80_ = 4.62, p < 0.01), but not treatment (data not shown). CRFR2 agonist-treated dams showed less nursing at t 0 min compared to the previous time point (p = 0.05).

*Other maternal behavior*: LG differed over time between the treatment groups (two-way RM ANOVA; F_8,80_ = 3.91, p < 0.01; Fig. 5A right). CRFR1 agonist-treated rats displayed significantly more pup LG after drug infusion at t +30 min (p < 0.01) and t +60 min (p = 0.02) compared with the VEH-treated group. No significant differences were observed for pup retrieval/mouthing (data not shown).

*Non-maternal behavior*: No differences were found for non-maternal behaviors, i.e. off-nest behavior (data not shown).

#### 3.3.2 Maternal care under stress conditions

No significant differences were found for ABN, nursing, pup retrieval/mouthing, and LG (data not shown).

#### 3.3.3 Maternal aggression

No differences were found in aggressive behaviors between the groups (data not shown).

#### 3.3.4 Oxytocin release

The mean basal oxytocin release was 2.83 ± 0.73 pg/sample in the VEH-, 2.88 ± 0.54 pg/sample in the CRFR1 agonist-, and 2.67 ± 0.31 pg/sample in the CRFR2 agonist-treated dams. There was a significant effect of time (two-way RM ANOVA; F_4,72_ = 4.19, p < 0.01) and treatment (F_2,18_ = 4.40, p = 0.02; Fig. 5B) on oxytocin release in the MPOA. Oxytocin release in the MPOA was significantly greater in the CRFR1 agonist-treated dams after drug infusion at t +30 min (p = 0.02) compared to the VEH-treated dams.

### 3.4 Experiment 4: Intra-MPOA CRFR1 agonist retrodialysis and oxytocin release in lactating rats

The mean basal oxytocin release was 1.73 ± 0.35 pg/sample in the VEH- and 1.70 ± 0.42 pg/sample in the CRFR1 agonist-treated dams. Oxytocin release in the MPOA changed significantly over time between the groups (two-way RM ANOVA; F_4,48_ = 3.40, p = 0.01; Fig. 5C). Oxytocin release was increased in the CRFR1 agonist-treated dams during (t 0 min: p < 0.01) and immediately after drug administration (t +30 min: p = 0.05), compared with the VEH group.

### 3.5 Experiment 5: Intra-MPOA inhibition of CRFBP in lactating rats

Acute infusion of a CRFBP inhibitor into the MPOA had no effect on maternal care (under non-stress or stress conditions), maternal motivation, maternal aggression, or anxiety-related behavior (data not shown).

## 4 Discussion

The brain CRF system is a potent regulator of maternal behavior. In brain areas such as the lateral septum (D'Anna and Gammie, 2009; Gammie et al., 2004) and the BNST (Klampfl et al., 2014, Klampfl et al., 2016a, Klampfl et al., 2016b) CRF plays a prominent role in impeding maternal behavior by reducing maternal care and maternal aggression while increasing anxiety. Thus, dampened CRFR activity in the peripartum period is crucial for high levels of maternal behavior and low maternal anxiety and hence, for successful rearing of the offspring.

Here, we advanced our current knowledge of the effects of an active CRF system in limbic brain areas to the hypothalamic MPOA, one of the most important brain regions for maternal behavior (Numan and Insel, 2003). We provide evidence that CRFR, particularly CRFR1, in the MPOA are involved in the regulation of maternal care, maternal aggression, and maternal anxiety inducing maternal neglect upon activation. Interestingly, *Crhr1* mRNA is predominantly expressed in the MPOA compared to *Crhr2*. However, we did not find a significant difference in expression between virgin and lactating rats suggesting that CRFR1 are not down-regulated postpartum as a potential adaptive mechanism to ensure reduced CRFR1 signal transduction.

Under non-stress conditions, ABN and total nursing were reduced following infusion of VEH or either CRFR agonist into the MPOA, which is consistent with our previous findings in the BNST suggesting that the infusion might be perceived as a mild stressor (Klampfl et al., 2014, 2016a). In addition, the behavioral profile following activation of MPOA CRFR is very similar to that observed following stimulation of anterior-dorsal BNST CRFR (Klampfl et al., 2016a). Indeed in both regions, CRFR1 stimulation strongly decreased ABN and nursing, while CRFR2 activation induced a more transient and less pronounced decline in ABN and nursing. However, it should be noted that the effect of the CRFR2 agonist in the MPOA on ABN may be artificial, as it is only different from VEH at t 0 min and t +30 min due to the greater levels of ABN under basal conditions in the VEH group. Nevertheless, both the CRFR1 and CRFR2 agonists significantly reduced total nursing behavior, and this effect was not a result of different levels of nursing under basal conditions. Moreover, the dams displayed significantly more non-maternal behaviors (i.e. off-nest behavior, locomotion, and self-grooming) over the period where ABN and nursing were reduced by the CRFR agonist treatments. Importantly, both CRFR antagonists prevented the infusion-induced reduction in ABN indicating a role for both receptors in the MPOA in the regulation of maternal care.

Under stress conditions, nursing was decreased while LG was increased in the CRFR1 agonist-treated dams. LG is generally considered a positive and beneficial behavior by the dam directed towards the pups (Champagne and Meaney, 2001), which is why an increase in LG by CRFR1 activation might seem counter-intuitive. However, CRF is well-known to induce self-grooming in both males and females (Dunn et al., 1987; Wiersielis et al., 2016), referred to as displacement activity (Kalueff et al., 2016). During lactation, this activity seems to be redirected from self-grooming to pup-grooming, thus increasing LG after stressor exposure. In support, a similar effect has been reported in lactating rats following exposure to white noise stress (Windle et al., 1997b). Moreover, inhibition of CRFR1 prevented the stress-induced reduction in ABN, but not nursing. Given that the rescuing effect of the CRFR1 antagonist is limited to ABN, it is likely that any stress-induced impairments in maternal care are mediated by CRFR in more stress-responsive brain regions, such as the BNST (Klampfl et al., 2014, 2016a).

Maternal motivation, as tested using the pup retrieval test, was not affected by CRFR manipulation, similar to findings following central and intra-BNST manipulations of CRFR in lactating rats (Klampfl et al., 2013, 2014, 2016a) and those in CRFR-deficient lactating mice (Gammie et al., 2007). The MPOA is one of the major brain regions involved in mediating maternal motivation (Numan and Insel, 2003; Numan and Woodside, 2010), however our finding that manipulating MPOA CRFR had no effect on this appetitive behavior further strengthens our general premise that the central CRF system is not involved in regulating maternal motivation postpartum.

During the maternal defense test, there was a robust increase in maternal aggression only in the dams administered the CRFR1 antagonist into the MPOA, indicating active CRFR1 during the maternal defense test and thus, a prominent role for MPOA CRFR1 in the regulation of maternal aggression. To our knowledge, this is the first time a direct involvement of the MPOA in modulating maternal aggression via a neurotransmitter system has been demonstrated. To date, the MPOA has only been reported to show increased neuronal activity upon an aggressive encounter in lactating mice (Gammie and Nelson, 2001) and rats (Motta et al., 2013). Furthermore, we are the first to demonstrate a direct effect of CRFR1 in regulating maternal aggression as previous studies revealed an exclusive role for CRFR2 in this behavior within the BNST (Klampfl et al., 2014) and lateral septum (D'Anna and Gammie, 2009) of rats, and in CRFR2-deficient mice (D'Anna et al., 2008).

In addition to maternal behavior, stimulation of CRFR1 activity within the MPOA had an anxiogenic effect. Indeed, the CRFR1 agonist, CRF, is well known to have anxiogenic actions in lactating females (Klampfl et al., 2013, 2014) and males (Koob and Thatcher-Britton, 1985). Interestingly, such an anxiogenic effect of CRF has not been demonstrated within the MPOA, and this brain region is not known to mediate anxiety-related behavior. However, in contrast to studies demonstrating an anxiolytic effect of ICV (Klampfl et al., 2013) or intra-BNST (Klampfl et al., 2014) CRFR1 blockade, inhibition of MPOA CRFR1 did not alter maternal anxiety. On the one hand, this discrepancy might be explained by a floor effect in the CRFR1 antagonist group as the dams' anxiety levels were generally very low. On the other hand, this could point to an indirect effect of CRFR1 activation on anxiety through reduced locomotion. Indeed, the MPOA is neuronally interconnected with the zona incerta and the pedunculopontine nucleus, brain areas that are involved in mediating locomotion (Swanson et al., 1987). Thus, decreased locomotion may result in less time spent on the open arms of the EPM, which is typically interpreted as increased anxiety, but may rather be an indirect consequence. Nevertheless, it remains possible that CRF's effect on anxiety seen here was a direct action, as ICV CRF infusion in novel environments (such as the EPM) can decrease general locomotor activity (Baldwin et al., 1991; Koob and Heinrichs, 1999). Hence, further studies are needed to closely delineate the effects of CRFR1 activation on maternal anxiety from those on general activity.

Since previous studies have demonstrated neuronal interactions between the CRF and oxytocin systems (Bosch et al., 2016; Dabrowska et al., 2011, 2013; Windle et al., 2004), we investigated whether an interaction between these two systems could shed light on the down-stream mechanism through which CRFR activation impairs maternal behavior. Given that oxytocin is known to promote the onset and maintenance of maternal care (Bosch and Neumann, 2012; Numan and Insel, 2003) and administration of an oxytocin receptor antagonist into the MPOA decreases ABN (Pedersen et al., 1994), we hypothesized that activation of central CRFR would reduce oxytocin release in the MPOA and hence impair maternal care. However, contrary to our prediction a single acute ICV infusion, as well as intra-MPOA retrodialysis, of a CRFR1 agonist increased oxytocin release in the MPOA under non-stress conditions. This seemed to be specific to the MPOA given that the effect of the CRFR1 agonist on oxytocin release was similar regardless of whether the drug was given centrally (experiment 3) or locally (experiment 4) into the MPOA. As oxytocin is released upon stressor exposure to reduce the hypothalamo-pituitary-adrenal axis response (Windle et al., 1997a), it is feasible that oxytocin is released upon CRFR1 activation in the MPOA to counteract CRF's effects on maternal behavior and ultimately loop back to regulate CRF neurons in the MPOA. Indeed, in the PVN oxytocin has been shown to reduce *Crh* mRNA (Bulbul et al., 2011; Windle et al., 2004) and stress-induced Fos expression (Windle et al., 2004), to delay the stress-induced rise in *Crh* transcription (Jurek et al., 2015), and to modulate CRF neuronal excitability (Jamieson et al., 2017). Moreover, stress-induced social buffering requires prolonged oxytocin release within the PVN, thereby inducing faster stress recovery in female prairie voles (Smith and Wang, 2014) and supporting a role for oxytocin in terminating the stress response. Thus, oxytocin release might be triggered by CRFR1 activation in the MPOA as a rescuing mechanism to minimize a reduction in maternal behavior and help provide stable levels of care for the offspring. However to date, little is known about neuronal interactions between the CRF and oxytocin systems in the MPOA. Even though CRF and oxytocin are co-expressed in some hypothalamic structures (Meister, 1993), neither cells nor fibres of these peptides co-localize in the MPOA, at least in male rats (Simerly and Swanson, 1988). Thus, it seems more likely that both CRF and oxytocin are not released upon the same stimulation from the same neuronal population, but rather that oxytocin is released after CRF has bound to and activated CRFR1. Similar interactions have recently been described in male prairie voles (Bosch et al., 2016; Pohl et al., 2018); activation of central CRFR2 suppresses, while inhibition of central CRFR2 increases, oxytocin release in the nucleus accumbens from neurons originating in the PVN. Further experiments investigating potential sites of CRFR1 and oxytocin co-expression would enhance our understanding of the mechanisms underlying the interactions between the CRF and oxytocin systems in the MPOA, and their impact on maternal behavior.

Given that *Crhr1* and *Crhr2* mRNA levels are unchanged and *Crh* mRNA expression is in fact elevated in lactation (Walker et al., 2001), we postulated that CRFBP, whose expression is increased by stress (Lombardo et al., 2001; McClennen et al., 1998; Westphal and Seasholtz, 2006), might be important for minimizing CRFR activity. However, we did not find any effects of inhibiting CRFBP in the MPOA on maternal or anxiety-like behaviors, neither did we detect any change in *Crhbp* mRNA expression during lactation that would support such a role. It is unlikely that the dose used was too low to fully inhibit CRFBP in the MPOA, as the same dose administered into the BNST elicits behavioral changes (Klampfl et al., 2016b), and there is no indication that CRFBP concentrations in the MPOA differ markedly from those in the BNST. Rather, it seems more likely that CRFBP in the MPOA is not involved in dampening the activity of the CRF system postpartum.

In conclusion, we report that activation of CRFR, particularly CRFR1, in the MPOA impedes maternal care and maternal aggression and increases maternal anxiety. Furthermore, CRFBP does not appear to be involved in the crucial down-regulation of the CRF system postpartum. Yet, we identified another potential regulatory mechanism involving oxytocin release in the MPOA, which may serve to counteract the effects of CRFR activation, thereby ensuring stable levels of maternal behavior. Interestingly, this mechanism might be disturbed in mothers showing stress-induced maternal neglect and represents a potential target for treating such malfunctions in the postpartum period.

## Declarations of interest

None.
